# The New Yeast Is a Mouse

**DOI:** 10.1371/journal.pbio.1000106

**Published:** 2009-05-05

**Authors:** Rhona H Borts

## Abstract

Recombination hotspots are determined not only by features of the local genome but also by sequences acting at a considerable distance both in*cis* and*trans*.

Sexual reproduction depends on a specialized type of cell division called meiosis to generate the sperm and egg cells (gametes) that fuse to form an embryo. Meiosis carries out two important functions: recombination, which generates the diversity on which evolution acts, and reduction of the chromosome number from the full complement (diploid) to half (haploid). Every somatic cell in the human body contains 23 pairs of chromosomes: one set from the mother and one set from the father. When these cells divide, every daughter cell gets one copy of each pair of chromosomes. However, if the gametes contained both sets of chromosomes when they combined during fertilization, the embryo would have twice the normal amount of genetic information. Meiosis (Figure 1) avoids this problem by ensuring that each gamete gets only one copy of each chromosome pair. When the correct partitioning of chromosomes fails (non-disjunction), parental infertility or offspring with an abnormal number of chromosomes result.

**Figure 1:**
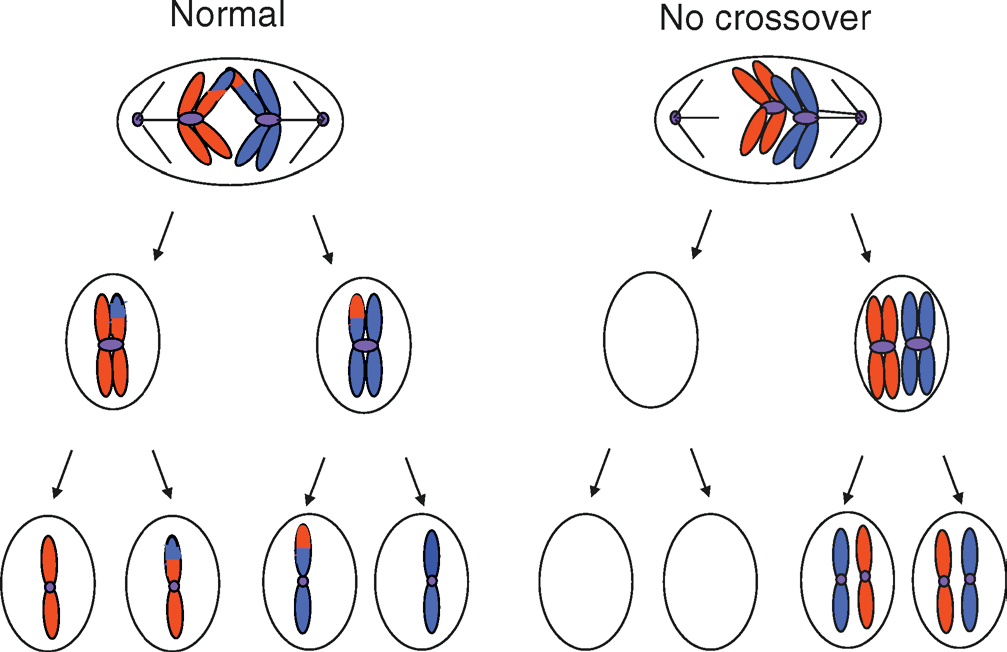
In the Absence of Crossing Over, Chromosomes Mis-Segregate Tension provided by the proteinaceous structures called chiasmata, which are associated with crossovers, allows the chromosomes to be oriented correctly at metaphase. In the subsequent division, each gamete gets one parental chromosome. In the absence of any crossovers, both chromosomes can be carried to the same pole of the cell. At the second meiotic division, this results in two gametes with twice the number of chromosomes than they should have. In humans, the majority of such gametes do not lead to viable progeny. However, this type of mis-segregation of Chromosome 21 in maternal meiosis leads to trisomy 21—or Down syndrome—when the oocyte is fertilized by a normal sperm.

In many organisms including yeast, mice, and humans, an essential feature of meiosis is genetic recombination. Recombination creates diversity by mixing the genetic information from each parent into new combinations. Recombination events can be either a reciprocal exchange of DNA called a crossover or a nonreciprocal exchange called a gene conversion or noncrossover (Figure 2). It is the crossovers that become part of a physical structure called chiasmata, which ensures that the homologous chromosomes go to opposite poles and thus partition properly. Because of this essential role, organisms have developed mechanisms (interference [1] and crossover homeostasis [2]) to distribute crossovers nonrandomly within and between chromosomes, such that each chromosome gets at least one crossover (the “obligate” chiasmata [3]). The molecular basis and the relationship between these mechanisms are poorly understood.

**Figure 2:**
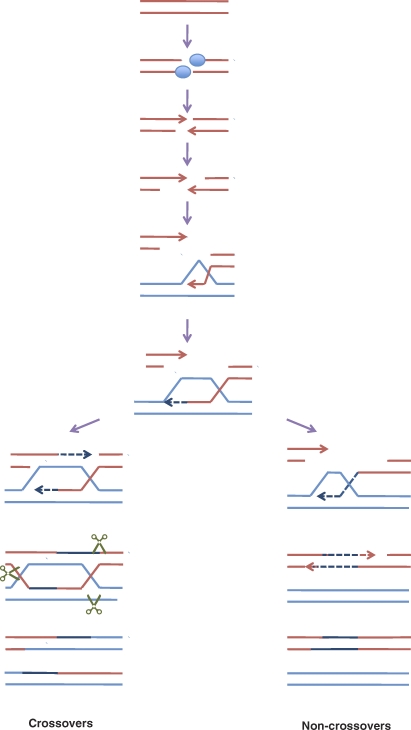
Recombination Is Initiated by Double-Strand Breaks Double-strand breaks are nonrandomly distributed at hotspots. Rcr1/Dsbc1 influence the frequency with which particular hotspots are broken. Once broken, an exonuclease creates single-stranded DNA that can then invade the other parental chromosome. Just prior to, or at this time, other factors influence whether the crossover or noncrossover repair pathway is used. On the crossover pathway, the strand invasion primes DNA synthesis that displaces the resident strand. This is captured by the single-stranded DNA on the other side of the double-strand break, forming a structure called a Holliday junction. This is thought to be resolved by the recently discovered Gen1/Yen1 protein [36], illustrated by the scissors. This pathway results in the exchange of chromosome arms as illustrated in Figure 1. In the noncrossover pathway, strand invasion primes DNA synthesis forming a migrating D-loop. The unwound DNA pairs with the opposite side of the break, and more synthesis and ligation create a noncrossover recombination product.

In all organisms analysed to date, recombination is initiated by a double-strand breaks in the DNA catalysed by a protein called Spo11 [4]. In many organisms (fruit flies and worms being exceptions), double-strand breaks do not occur randomly but are more frequent in very small regions of the genome called hotspots (Figure 3A). The activity of these hotspots is highly variable, ranging over a few orders of magnitude. However, bringing Spo11 to DNA is not always sufficient to initiate recombination [5]. What else does it take? How and why a hotspot is hot is one of the burning issues to those studying the mechanisms of meiotic recombination. Understanding the molecular mechanisms of distribution of crossovers is important for many reasons, among which are the impact that hotspots have on where diversity can and cannot be generated and the effect they have on disease association studies. In some organisms, there appears to be sequence specification of some but not all hotspots [6,7]. In the yeast Saccharomyces cerevisiae, double-strand breaks occur within a few hundred base pairs of transcriptional start sites. However, only some of these hotspots (alpha hotspots) can be shown to be activated by transcription factors. The well characterized hotspot located within the promoter of the gene *HIS4* is dependent on the transcription factors Bas1/Bas2 [8], Rap1 [9], and Gcn4 [10] for full recombination activity and contains binding sites for all of these factors. Ablation of the transcription factor binding sites abolishes recombination. Both the Bas1/Bas2 [11] and Gcn4 [12] transcription factors are essential for response to various starvation and stress signals, indicating that there are complex links between the external environment and crossover frequencies. Linking the genome-wide shuffling of genetic information to changes in the environment could be highly adaptive for a microorganism. The fission yeast Schizosacharomyces pombe also uses transcription factors to initiate some recombination. One of the best-characterized hotspots is the M26 allele of the *ade6* gene [6]. This mutation dramatically increased recombination at the *ade6* locus. It has been shown to be a binding site for the stress response transcription factor Atf1-Pcr1 and, as in S. cerevisiae, recombination is dependent on this transcription factor. However, the transcription factor binding sequence is not sufficient for hotspot activity even when the Atf1/Pcr1 is present, indicating that other chromosomal features are necessary [6]. All yeast hotspots are contained in regions of open chromatin [13]. The opening of this chromatin is also dependent on histone modifications [14]. Recent work in yeast has suggested that particular histone modifications actually mark potential hotspots before meiosis [15]. Thus, recombination frequency is not just a feature of local DNA sequence but in fact depends on factors encoded elsewhere in the genome.

**Figure 3:**
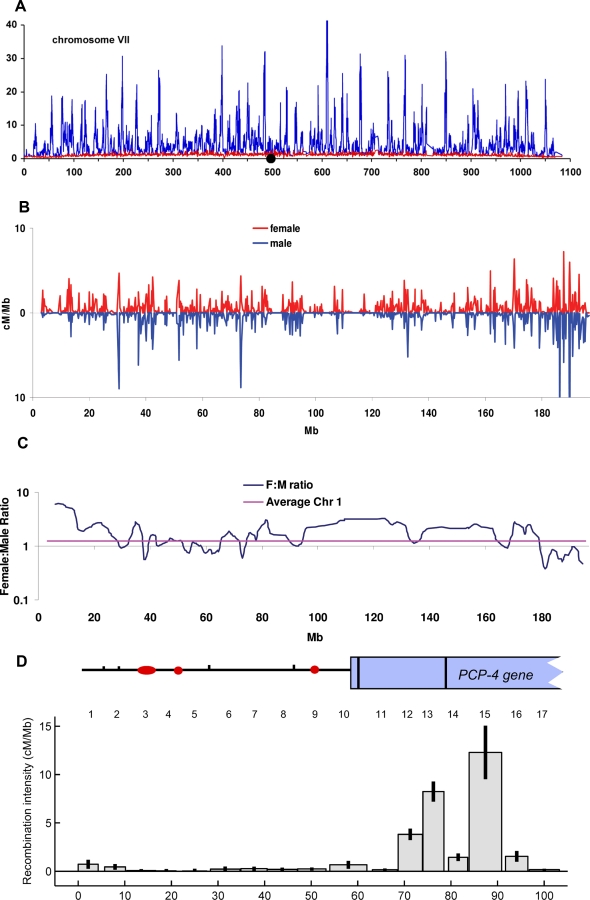
Nonrandom Distributions of Double-Strand Break Hotspots in Yeast and Crossover Hotspots in Mice and Humans (A) illustrates the distribution of double-stand breaks on a single chromosome in yeast [37]. A recent genome-wide crossover map made in a hybrid yeast diploid [38] shows a good, but not perfect, correlation between double-strand breaks and crossover hotspots, suggesting that not all double-strand break hotspots are crossover hotspots. (B) illustrates differences in the distribution of crossovers on Chromosome 1 in male and female meioses [20]. One can see that, in general, the same hotspots are used but that the intensity varies in males and females. This can be clearly seen in (C), where the ratios of crossovers in females to males is plotted across the chromosome. (D) illustrates the punctate distribution of crossovers in a small region of human Chromosome 21 as measured by sperm typing analysis [39].

Recombination frequencies are modulated by genetically determined factors in higher organisms as well. In humans, there is variation of total crossover frequencies from individual to individual [16], whereas inbred mice show differing frequencies of recombination [17]. There are also differences between male and female mammals in both the number and distribution of crossovers. This has been demonstrated cytologically by counting foci for an essential recombination protein, Mlh1, that marks sites of crossing over in both male and females [18], as well as by pedigree analysis [19] and by crossover hotspot mapping [20] (Figure 3B). Individual human males can be shown to vary by direct measurement of crossing over at specific hotspots [21,22]. This variation can, in some cases, be attributable to sequence variation at the hotspot [23], as has been seen in yeasts when transcription factor binding sites at hotspots are mutated. In other cases, there are no obvious sequence differences at the hotspot, indicating that sequence nonspecific factors [19] and/or distal elements as seen in the mouse [24,25] (discussed below) can influence recombination rate [22]. That there are genetically determined *trans*-acting factors can be seen from the elegant work of Coop et al. [26] and Kong et al [27]. Coop et al. demonstrated that there were heritable differences in recombination frequencies in families, while Kong et al. mapped such a difference to a polymorphism in the *RNF12* gene. Interestingly this polymorphism is associated with high rates of recombination in males and lower rates in females, indicating that sex-specific factors influence its activity. Indeed, a colleague and I suggested that sex-specific hormonal control of transcription factors might account the differing patterns of recombination in male and female meiosis [10], although hotspots in humans do not appear to map to promoter regions. Recent work from the Hunt lab has shown that chemically (Bisphenol) or genetically (targeted disruption) interfering with an estrogen receptor (ERβ) in mouse affects crossover frequencies (among many other things) in mouse oocytes [28].

Unfortunately, humans are not an experimentally tractable organism and yeasts are not mammals. And indeed one of the catch 22's of recombination studies is that while sequence divergence and genetic diversity are necessary to study recombination, they themselves can influence the outcome [29]. Thus, in humans, any study of recombination may be influenced by the sequence polymorphisms used to measure the event and by the 1,000s of potential genetic differences amongst individuals. In yeast, researchers have been be able to generate isogenic strains that differ only in the markers they wish to study and in specific genetic controlling elements. Fortunately, recent technical advances in high-throughput SNP detection and directed breeding, as well as in cytology and single-molecule recombination analysis, have made it possible to do very elegant and sophisticated recombination analysis in mice. The new yeast is a mouse. The experiments presented in Grey et al. [25] and Parvanov et al. [24] in the February 2009 issue of *PLoS Biology* identify a region of DNA that, when derived from a particular mouse strain, stimulates crossing over at hotspots that are located megabases away on the same chromosome, while repressing others and also acting on hotspots located on different chromosomes. This research identifies *trans* -acting factors in an experimentally tractable mammalian system. These two groups, using two very different experimental approaches, have defined a region of 5.3–6.7 megabases whose genotype can influence crossing over genome wide. This factor modifies the distribution of crossovers thus altering the genetic map with no loss of over overall crossing over.

The group of de Massy has extensively analysed recombination at the Psmb9 locus using single-molecule crossover and conversion assays [30,31,32]. When one parental chromosome in the crossover assay carries a Chromosome 17, derived in part from Mus musculus molossinus [33] (wm7 haplotype), recombination is stimulated in both *cis* and *trans*. By using sequence polymorphisms between two mouse backgrounds to map crossovers, the researchers defined the hotspot as a narrow region where recombination is initiated [32]. They further showed that hotpot activity was dependent on Spo11 demonstrating that double-strand breaks are the initiating event. They further showed that crossover recombination is dependent on the *Mlh1* [32] and *Mlh3* [30] genes as it is known to be in S. cerevisiae [34,35]. The region responsible for hotspot activity has been found to be located in a relatively small region located approximately 20 megabases away from the hotspot. This clearly indicates that it is a *trans*-acting factor that they have named Dsbc1 (double-strand break control 1).

The Paigen group used an altogether different approach of interstrain crosses between a wild mouse Mus musculus casteneus (CAST/EiJ) and a laboratory mouse to specifically search for *trans*-acting loci affecting recombination on Chromosome 1. They found a 5.3-megabase region from the CAST/EiJ mouse that is contained within the region on Chromosome 17 found by Grey et al [25]. They have called their region “Recombination regulator 1” (Rcr1). Although it is not definitive proof that the *trans*-acting factors are the same, it is interesting to note that the source of the sequence in the wm7 haplotype could be Mus musculus casteneus.

Both groups have demonstrated that both crossovers and noncrossovers are affected, indicating that the factor(s) influence initiation. Both groups have found that some hotspots are stimulated, some suppressed, and others not affected at all, indicating, not surprisingly, that the control of recombination is complex, as in the yeasts. How Dsbc1/Rcr1 acts is not clear. Both groups speculate that it is influencing chromatin structure, although the Paigen group argue that the regions affected are small, since two very close hotspots are differentially affected. The identification the gene(s) encoding these factors will hopefully shed light on the mechanism of action and contribute greatly to our understanding of the regulation of the important process of meiotic crossing over.
